# S224. DELTA-9-TETRAHYDROCANNABINOL CHALLENGE IN CANNABIS USERS AND NON-USERS DIFFERENTIALLY AFFECTS BRAIN FUNCTION AND BEHAVIOR: AN FMRI STUDY OF DEVELOPMENT OF TOLERANCE

**DOI:** 10.1093/schbul/sby018.1011

**Published:** 2018-04-01

**Authors:** Marco Colizzi, Philip McGuire, Vincent Giampietro, Steve Williams, Mick Brammer, Sagnik Bhattacharyya

**Affiliations:** Institute of Psychiatry, King’s College

## Abstract

**Background:**

Cannabis use can induce acute and long-lasting psychosis and cognitive dysfunction. Some evidence suggests that the acute behavioral and neurocognitive effects of the main active ingredient in cannabis, (−)-trans-Δ9-tetrahydrocannabinol (∆9-THC), might be modulated by previous cannabis exposure. However, this has not been investigated either using a control group of non-users, or following abstinence in modest cannabis users, who represent the majority of recreational users.

**Methods:**

Twenty-four healthy men participated in a double-blind, randomized, placebo-controlled, repeated-measures, within-subject, ∆9-THC challenge study.

**Results:**

Compared to non-users (N=12; <5 lifetime cannabis joints smoked), abstinent modest cannabis users (N=12; 24.5 ± 9 lifetime cannabis joints smoked) showed worse performance and stronger right hemispheric activation during cognitive processing, independent of the acute challenge (all P≤0.047). Acute ∆9-THC administration produced transient anxiety and psychotomimetic symptoms (all P≤0.02), the latter being greater in non-users compared to users (P=0.040). Non-users under placebo (control group) activated specific brain areas to perform the tasks, while deactivating others. An opposite pattern was found under acute (∆9-THC challenge in non-users) as well as residual (cannabis users under placebo) effect of ∆9-THC. Under ∆9-THC, cannabis users showed brain activity patterns intermediate between those in non-users under placebo (control group), and non-users under ∆9-THC (acute effect) and cannabis users under placebo (residual effect). In non-users, the more severe the ∆9-THC-induced psychotomimetic symptoms and cognitive impairments, the more pronounced was the neurophysiological alteration (all P≤0.036).

**Discussion:**

Previous modest cannabis use blunts the acute behavioral and neurophysiological effects of ∆9-THC, which are more marked in people who have never used cannabis.

